# Bioinformatics and System Biological Approaches for the Identification of Genetic Risk Factors in the Progression of Cardiovascular Disease

**DOI:** 10.1155/2022/9034996

**Published:** 2022-08-09

**Authors:** Joy Dip Barua, Shudeb Babu Sen Omit, Humayan Kabir Rana, Nitun Kumar Podder, Utpala Nanda Chowdhury, Md Habibur Rahman

**Affiliations:** ^1^Department of Pharmacy, BGC Trust University Bangladesh, Chattogram, Bangladesh; ^2^Department of Computer Science and Telecommunication Engineering, Noakhali Science and Technology University, Noakhali, Bangladesh; ^3^Department of Computer Science and Engineering, Green University of Bangladesh, Dhaka, Bangladesh; ^4^Bangladesh Institute of Governance and Management (BIGM), Dhaka, Bangladesh; ^5^Department of Computer Science and Engineering, Khulna University of Engineering & Technology, Khulna, Bangladesh; ^6^Department of Computer Science and Engineering, University of Rajshahi, Rajshahi, Bangladesh; ^7^Department of Computer Science and Engineering, Islamic University, Kushtia 7003, Bangladesh

## Abstract

**Background:**

Cardiovascular disease (CVD) is the combination of coronary heart disease, myocardial infarction, rheumatic heart disease, and peripheral vascular disease of the heart and blood vessels. It is one of the leading deadly diseases that causes one-third of the deaths yearly in the globe. Additionally, the risk factors associated with it make the situation more complex for cardiovascular patients, which lead them towards mortality, but the genetic association between CVD and its risk factors is not clearly explored in the global literature. We addressed this issue and explored the linkage between CVD and its risk factors.

**Methods:**

We developed an analytical approach to reveal the risk factors and their linkages with CVD. We used GEO microarray datasets for the CVD and other risk factors in this study. We performed several analyses including gene expression analysis, diseasome analysis, protein-protein interaction (PPI) analysis, and pathway analysis for discovering the relationship between CVD and its risk factors. We also examined the validation of our study using gold benchmark databases OMIM, dbGAP, and DisGeNET.

**Results:**

We observed that the number of 32, 17, 53, 70, and 89 differentially expressed genes (DEGs) is overlapped between CVD and its risk factors of hypertension (HTN), type 2 diabetes (T2D), hypercholesterolemia (HCL), obesity, and aging, respectively. We identified 10 major hub proteins (FPR2, TNF, CXCL8, CXCL1, IL1B, VEGFA, CYBB, PTGS2, ITGAX, and CCR5), 12 significant functional pathways, and 11 gene ontological pathways that are associated with CVD. We also found the connection of CVD with its risk factors in the gold benchmark databases. Our experimental outcomes indicate a strong association of CVD with its risk factors of HTN, T2D, HCL, obesity, and aging.

**Conclusions:**

Our computational approach explored the genetic association of CVD with its risk factors by identifying the significant DEGs, hub proteins, and signaling and ontological pathways. The outcomes of this study may be further used in the lab-based analysis for developing the effective treatment strategies of CVD.

## 1. Introduction

Cardiovascular disease is a leading cause of death in both developing as well as developed countries; by 2030, it is estimated that approximately 23. 6 million people will die of CVD [1]. According to the World Health Organization, four out of five CVD patients die from a heart attack or myocardial infarction every year. Cardiovascular disease has now become one of the leading causes of death and disability around the world. Moreover, the situation of cardiovascular disease is rapidly deteriorating because the risk factors of it are making the issue more complicated.

A number of research studies have been done for finding out the risk factors of CVD. Winkleby et al. found that smoking, systolic and diastolic blood pressures, and high-density lipoprotein cholesterol are risk factors for CVD [2]. Chiazor et al. observed that T2D, HTN, dyslipidemia, and smoking are responsible for CVD [3]. Smoking, diabetes mellitus, HTN, dyslipidemia, and HIV infection are reported by Friis-Møller et al. as risk factors [4]. Mozaffarian et al. said that T2D, HTN, and dyslipidemia are well-established predictors of cardiovascular disease [5]. Aging, HTN, HCL, obesity, T2D, smoking, and many other conditions are investigated as risk factors by Al Rawahi et al. [6]. Studies also show how these risk factors drive CVD patients towards mortality. T2D increases the glucose level in the blood, which reduces the blood vessels' elasticity and, as a result, impedes blood flow [7]. Similarly, HCL narrows the blood vessels and blocks blood flow that is the main cause of CVD [8]. HTN is also known as a risk factor for atherosclerosis development which results in coronary heart disease [9]. Atherosclerosis includes atrial enlargement link obesity to CVD [10]. Furthermore, aging is an independent risk factor for cardiovascular diseases [11].

Several transcriptomic and genetic studies were conducted using gene expression profiling, which revealed several genes that are differentially expressed in CVD, but the majority of these studies were constrained at the transcript level since there were no general considerations about functional associations between gene products and the effects of influencing factors. Many of the molecular mechanisms, gene functions, cell physiological interactions, and genetic profile of CVD are not fully explored yet. Therefore, we developed a computational approach to identify the potential biomarkers and biomolecules of CVD using gene expression microarray technology.

At present, gene expression analysis has become a popular tool for researchers to discover and investigate biomolecules, their mechanisms, and gene expression levels using microarray [12]. It is a technique that tells us which genes in a particular cell or tissue are turned on or off by determining the activity of thousands of genes [13]. By measuring its relative amounts of mRNA, gene expression profiling can also be used to determine the transcriptional level pattern in the genes expressed by a cell [14]. Again, microarray technology helps to detect gene expression by comparing disease and healthy mRNA samples [15]. In our research, we used publicly available microarray datasets for the gene expression profiling to detect the genetic risk factors of CVD.

In this study, we first identified the DEGs of CVD and our selected risk factors. After that, we did cross-comparative analysis, PPI analysis, pathways analysis, and gene ontological analysis. We identified significant hub proteins, functional and ontological pathways using the shared DEGs of CVD, and its risk factors. We further applied a network-based analysis to understand the gene activity relationships between CVD and its risk factors at the molecular level. Finally, we validated our research work through gold benchmark databases that indicates that our identified risk factors may be the potential risk factors of CVD.

## 2. Materials and Methodology

### 2.1. Brief Description of the Analytical Approach

**Figure 1:**
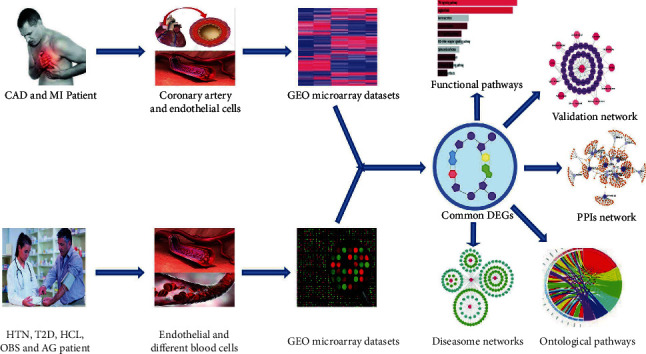
Block diagram of the applied analytical approach.

In our analytical approach, we first identified the DEGs of CVD and its risk factors employing the gene expression microarray data. After that, we did cross-comparative analysis to find the shared DEGs between CVD and its risk factors. We then identified significant hub proteins and functional and ontological pathways using those shared DEGs. We further applied a network-based analysis to understand the gene-disease association ship of CVD and its risk factors at the molecular level. Finally, we validated our study through the gold benchmark databases and found our selected four risk factors as the potential risk factor of CVD. The pictorial representation of our analytical approach is shown in Figure 1.

### 2.2. Dataset Description

We surveyed globally published literature and found some links among CVD and HTN, T2D, HCL, obesity, and aging from clinical studies. Then, we collected Gene Expression Omnibus (GEO) datasets from National Centre for Biotechnology Information (NCBI) (https://www.ncbi.nlm.nih.gov/geo/), a publicly available repository. We examined a number of available microarray datasets, but most of them were discarded because of low number of samples or absence of cases, controls, and gene symbols. Also, we did not consider the datasets that were not generated from human organisms. After considering all the criteria, we collected 7 microarray datasets for CVD, HTN, T2D, HCL, obesity, and aging where for cardiovascular disease, we considered coronary artery disease and myocardial infarction datasets. Accession numbers for HTN, T2D, HCL, obesity, aging, coronary artery disease, and myocardial infarction are GSE703, GSE26168, GSE6054, GSE60403, GSE13712, GSE98583, and GSE66360, respectively. The microarray dataset of HTN is derived from peripheral white blood cells of 6 healthy individuals and 14 individuals with pulmonary arterial hypertension [16]. T2D dataset is a gene expression array that was generated from the blood samples of 8 healthy people and 16 patients using the Illumina technology [17]. The HCL dataset was derived by comparing 13 controls and 10 familial hypercholesterolemia participants using monocyte cells from their blood which is also a microarray dataset used Affymetrix technology [18]. The microarray dataset of obesity was developed using Affymetrix technology from the blood of human umbilical cord; there were 8 obese and 8 leans individuals [19]. The microarray dataset of aging was extracted from the human umbilical vein of 6 young and 6 senescent people using the Affymetrix platform [20]. Both cardiovascular microarray datasets were extracted from human blood samples where coronary artery disease dataset was produced by comparing 6 healthy with 12 case samples [21], while the myocardial infarction dataset was developed using 50 healthy people and 49 infected subjects [22] using the same Affymetrix technology. A brief description of the datasets is given in Table 1.

### 2.3. Analysis Methods

Gene expression data analysis is a worldwide popular and effective technique for exploring the genetic profile at the molecular level [23]. We applied the gene expression analysis technique on the CVD and its risk factors to investigate the biological profile and their associations at the genomic level. As our selected mRNA expression data are produced from different technologies and experimental setups, we minimized the complications by normalizing the original datasets using *Z*-score transformation (*Z*_*ij*_) for each of the risk factor and their main malady CVD as follows:

(1) $$\begin{array}{c} Z_{ij}=\frac{x_{ij}–{\underline{x}}_{i}}{\sigma_{i}}, \end{array}$$

where *σ* denotes the standard deviation, $\underline{x}$ denotes the mean, and *x*_*ij*_ denotes the gene expression value *i*  in *j* sample. As a result of this *Z* transformation, different disease stages can be appropriately compared with each other in terms of gene expression. For detecting differentially expressed genes, we applied the unpaired Student's *t*-test statistic along with the threshold *p* value ≤ 0.05 and |logFC| ≥ 1 to each malady control dataset, which identified significantly dysregulated genes (up- and downregulated genes).

To achieve gene-disease interaction and correlation, we constructed a gene-disease network or bipartite graph using neighborhood-based benchmark and multilayer topological strategies for the gene-disease associations, where the nodes of the network are either gene or disease. The main condition for diseases to participate in the gene-disease network is that the diseases must share one or more significant dysregulated genes among them. Let *D* be the set of diseases and *G* is be the set of dysregulated genes, and gene-disease affiliations are determined by whether gene *g* ∈ *G* is associated with disease *d* ∈ *D*. If the set of significant dysregulated genes *G*_*i*_ and *G*_*j*_ is linked with diseases *D*_*i*_ and *D*_*j*_, respectively, after that, the equation of the number of dysregulated genes (*n*_*ij*_^*g*^), shared by both diseases *D*_*i*_ and *D*_*j*_, can be expressed as follows [24, 25]:

(2) $$\begin{array}{c} n_{\text{i}j}^{g}=N\;\left(G_{i}\cap G_{j}\right). \end{array}$$

The Jaccard coefficient method is used to select the common neighbors by measuring the edge prediction score for the node pair according to their similarity as follows [26, 27]:

(3) $$\begin{array}{c} \text{E}\left(\text{i},\text{j}\right)=\frac{N\;\left(G_{i}\cap G_{j}\right)\;}{N\;\left(G_{i}\cup G_{j}\right)}, \end{array}$$

where *G* and *E* represent the set of nodes and set of all edges, respectively.

For the protein-protein interaction analysis, STRING [28] database is utilized, which is an online tool for the retrieval of interacting genes or proteins. Besides, we utilized a network visualization tool named Cytoscape [29] for the visualization of the network where nodes denote proteins and edges denote the connections between the nodes. Additionally, closely linked nodes, i.e., hub proteins, are identified applying the degree metrics by the cytoHubba [30] plugin of Cytoscape.

For a deeper understanding of biological functions and pathways, a web-based gene-enrichment analysis platform EnrichR [31] is used to analyze the pathways and gene ontologies for the common DEGs of CVD and its risk factors. For pathway enrichment analysis, Kyoto Encyclopedia of Genes and Genomes [32], Reactome [33], and WikiPathways [34] human databases were used to obtain the significant enrichment outcomes. Similarly, GO biological process [35], GO molecular function [35], and GO cellular component [35] databases were used for ontological analysis where the threshold of selecting both pathways and ontologies was *p* value ≤ 0.05.

To verify the relationship of CVD with its risk factors and to evaluate the utility of the network-based approach, three standard gold benchmark databases OMIM [36], dbGAP [37], and DisGeNET [38] were used. We collected diseases with its associated genes from the above-mentioned databases using DEGs of CVD. After applying several statistical analyses, we shortlisted the collected diseases and found our selected four risk factors in the list.

Step-by-step instructions for the analytical approach are as follows:

(1) Search datasets in the public repositories by considering necessary standards
(2) Datasets, *i* = 1, 2, ⋯, *N*, were obtained using the follow steps
  - Load dataset
  - Make column names
  - Group all samples
  - Apply log2 transformation
  - Apply normalization to the data
  - Assign patient's samples to case and healthy people's samples to the control group
  - Set up case vs. control design matrix
  - Apply precision weights
  - Fit linear model
  - Recalculate coefficient
  - Generate statistics and table with Benjamini and Hochberg
  - Download the data table and delete empty gene rows
(3) For differentially expressed genes
  - Apply *p* value and logFC value
  - Apply false discovery rate (FDR)
  - List identified DEGs
(4) Contrast of two distinct diseases of DEGs for
  - overlapping upregulated genes
  - overlapping downregulated genes
(5) Utilizing common up- and downregulated DEGs for
  - construction of gene-disease association network
  - protein-protein interaction analysis
  - hub protein identification
  - signaling pathway enrichment analysis
  - ontological pathway enrichment analysis
(6) Validation of the work using gold benchmark databases

## 3. Results

### 3.1. Differentially Expressed Gene Identification

For the investigation on risk factors and the genomics of CVD progression, we examined GEO microarray datasets from National Center for Biotechnology Information (NCBI). We employed limma [39], umap [40], and GEOquery [41] R packages of GEO2R [42] tool to obtain gene expression scores by comparing disease and normal samples. We identified DEGs consisting of up- and downregulated genes where upregulated genes are selected using the threshold *p* value ≤ 0.05 and logFC ≥ 1 and downregulated genes using *p* value ≤ 0.05 and logFC ≤ −1. We detected a total number of 201 DEGs for HTN where the number of up- and downregulated genes are 122 and 79. In T2D, 233 up- and 339 downregulated genes are identified where total DEGs are 572. 1195 DEGs were identified for HCL including 538 up- and 657 downregulated genes. For obesity, 816 up- and 561 downregulated genes were found, and the total numbers of DEGs are 1377. A total of 2693 DEGs were determined for aging, which consisted of 1046 up- and 1647 downregulated genes. We identified 534 and 951 DEGs for coronary artery disease and myocardial infarction, respectively, where up- and downregulated genes in coronary artery disease are 87 and 477, and for myocardial infarction, the numbers are 718 and 233. The summary of the identified DEGs is shown in Table 2.

**Figure 2:**
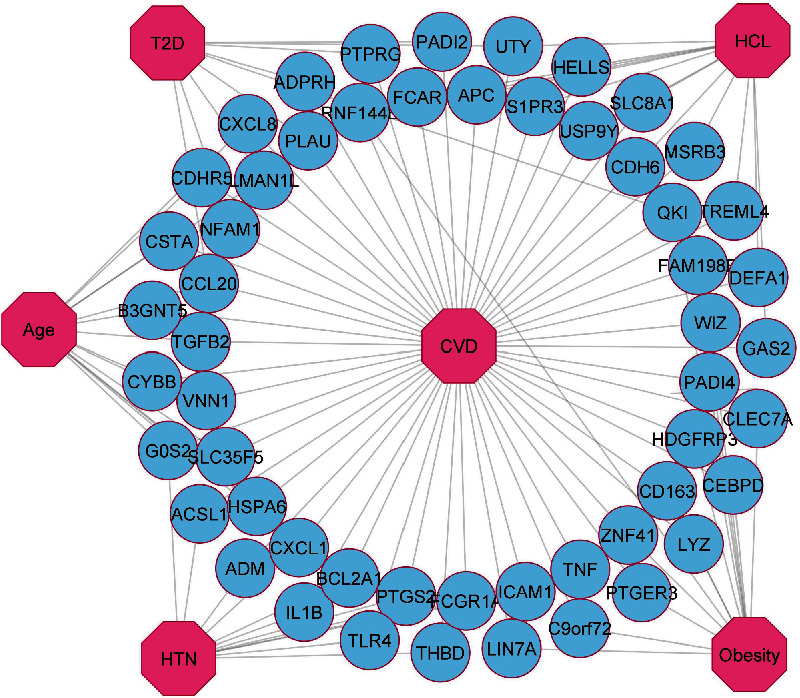
Diseasome network of the upregulated genes that are common between CVD and HTN, T2D, HCL, obesity, and aging. Circular sky blue-colored nodes represent shared upregulated genes, and octagon-shaped nodes represent CVD and its risk factors.

**Figure 3:**
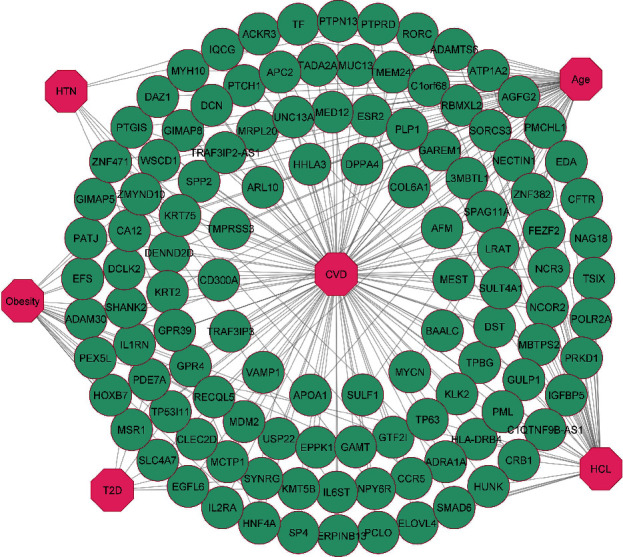
Diseasome network of the downregulated genes that are common between CVD and HTN, T2D, HCL, obesity, and aging. Circular green-colored nodes represent shared downregulated genes, and octagon-shaped nodes represent CVD and its risk factors.

There must be several DEGs in the two diseases for them to be associated [43]. Therefore, we performed a cross-comparison analysis on the identified DEGs among the CVD and the risk factors. We found that the shared DEGs of CVD with HTN, T2D, HCL, obesity, and aging are 32 (25 up and 7 down), 17 (10 up and 7 down), 53 (14 up and 39 down), 70 (47 up and 23 down), and 89 (39 up and 50 down), respectively. To exert the significant affinity, gene-disease association networks were constructed and presented in Figures 2 and 3, where common up- and downregulated genes were used between the risk factors and CVD. Our research also revealed that few genes are shared in CVD and more than one risk factor. Among them, the upregulated gene BCL2A1 is common in CVD, HTN, obesity, and aging; RNF144B and PTGIS are common in HCL, obesity, aging, and CVD; G0S2, CXCL1, ACKR3, and PTPRD are found in HTN, aging, and CVD; TGFB2, CA12, and MSR1 are familiar in obesity, aging, and CVD. Accordingly, S1PR3, IQCG, and TF are common in HCL, aging, and CVD; ACSL1, THBD, and HNF4A are repeated in HTN, obesity, and CVD. Similarly, CCL20 and PLAU are common in T2D, aging, and CVD, and UTY is in T2D, HCL, and CVD. Again, 3 upregulated genes ADPRH, PADI2, and QKI are shared in T2D, obesity, and CVD. The study also identified 3 additional genes CLEC7A, MSRB3, and PEX5L that are common among HCL, obesity, and CVD. In order to detect a significant correlation between CVD and risk factors, a biological network was constructed using the common DEGs of CVD, and its maladies are shown in Figures 2 and 3.

### 3.2. Protein-Protein Interactions and Hub Protein Identification

**Figure 4:**
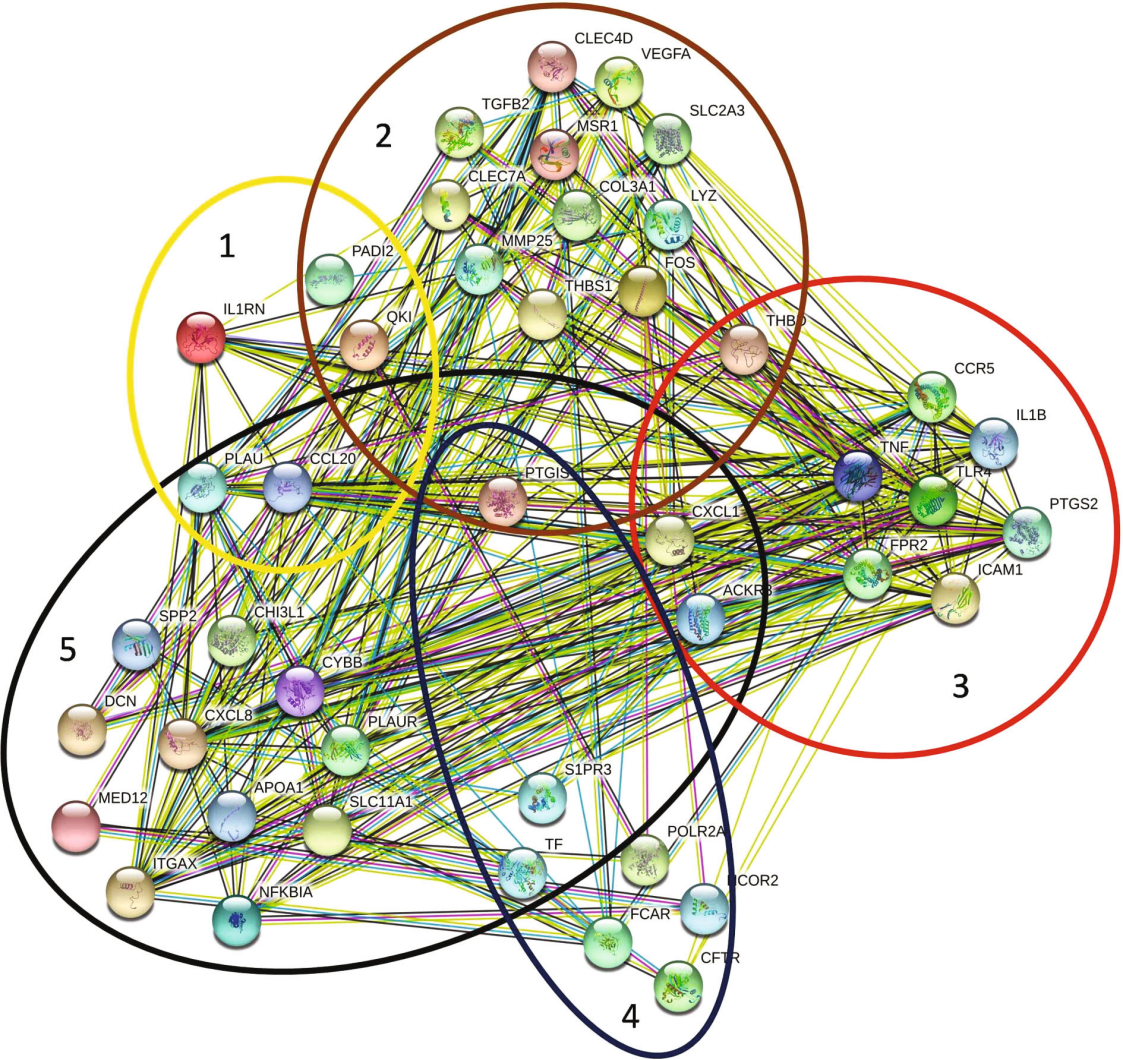
Protein-protein interaction (PPI) network of overlapping DEGs of CVD and its risk factors. The clusters indicate the proteins of the risk factors of CVD (elliptical shape clusters 1, 2, 3, 4, and 5 represent proteins of T2D, obesity, HCL, HTN, and aging, respectively) where some proteins are overlapped in more than one cluster.

Biochemical and biological functions of the proteins in a cell is known as protein-protein interaction. To understand cell physiology, drug design, and disease association, the investigation of protein-protein interaction contributes significantly in the field of biology and bioinformatics. Therefore, a PPI network is constructed utilizing the commonly identified 118 DEGs from a total of 213 DEGs among CVD and the selected five risk factors as shown in Figure 4. The nodes in PPI network denote proteins, and the undirected edges indicate the association between proteins. In addition, the network is divided into 5 clusters for the representation of protein's interactions of the risk factors and CVD. “PTGIS” belongs to the greatest number of clusters (3 clusters) that interact with other proteins of the other clusters. As a gene, it is also frequently identified in CVD, HTN, HCL and obesity. Besides, proteins including QKI, PADI2, CCL20, PLAU, THBD, CLEC7A, TF, S1PR3, CXCL1, and ACKR3 are all part of two clusters, each of which interacts with others in this network.

**Figure 5:**
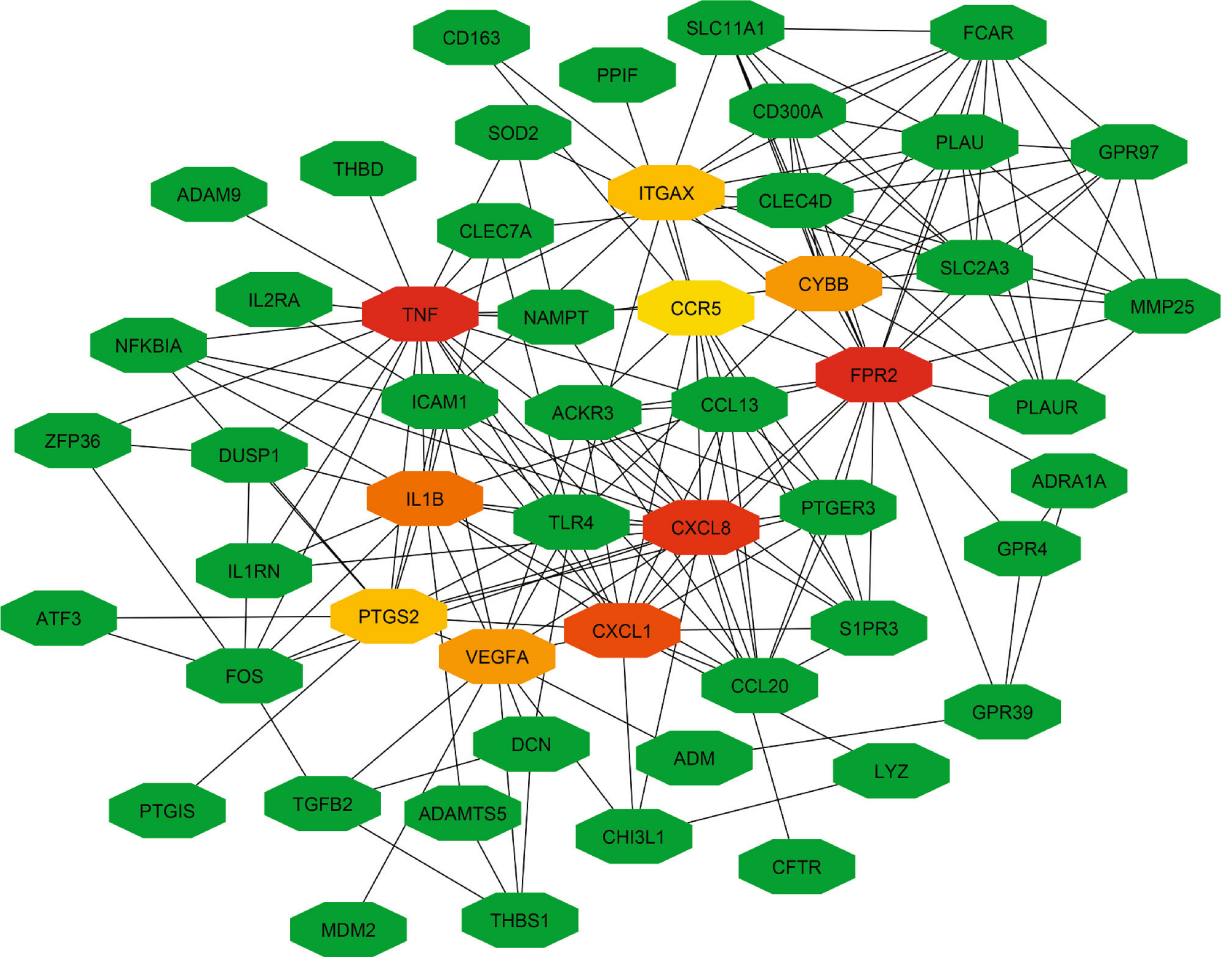
An illustration of the simplified PPI network and the hub proteins. Ten most significant hub proteins are marked as red, orange, and yellow color, respectively. Red color indicates highly connected nodes, orange color indicates moderately connected nodes, and yellow color indicates poorly connected nodes.

Hubs are nodes that possess a large number of links in a complex network, and hub proteins are proteins that have a significant number of interactions. Hub proteins are essential for the physiological interactions and drug design [44, 45]. We identified 10 major significant hub proteins (FPR2, TNF, CXCL8, CXCL1, IL1B, VEGFA, CYBB, PTGS2, ITGAX, and CCR5) from the PPI network using the cytoHubba plugin where 6 hub proteins are dysregulated for HTN, three for aging, and one for obesity. No hub proteins were found for the dysregulation of T2D and HCL. Detailed summaries of the hub proteins along with descriptions are given in Table 3, and the PPI network of hub proteins is depicted in Figure 5.

### 3.3. Identified Functional and Ontological Pathways

In order to understand the insights and how the diseases interact at the molecular level, it is essential to observe pathway-based analysis. A pathway also reveals internal changes in an organism as well as it may activate or disable genes. In this study, we performed functional pathway analysis to investigate molecular pathways among CVD and its risk factors by utilizing KEGG, Reactome, and WikiPathways databases of Enrichr. Based on the statistical significance (*p* value < 0.05) and the global published literature, we identified important pathways which have direct or indirect relationships with CVD and the risk factors. We found “fluid shear stress and atherosclerosis” (ID: hsa05418), “cardiac muscle contraction” (ID: hsa04260), “rheumatoid arthritis” (ID: hsa05323), “cytokine-cytokine receptor interaction” (ID: hsa04060), “TNF signaling pathway” (ID:hsa04668), “IL-17 signaling pathway” (ID:hsa04657), “chemokine signaling pathway” (ID: hsa04062), “TGF-beta signaling pathway” (ID: hsa04350), “MAPK signaling pathway” (ID: hsa04010), “NF-kappa B signaling pathway” (ID: hsa04064), “AGE-RAGE signaling pathway” (ID: hsa04933), and “PI3K-Akt signaling pathway” (ID: hsa04151) as effective pathways associated with CVD.  Table 4 summarizes the most significant identified pathways.

Gene ontologies represent biological information such as gene functions, gene relationships, and gene attributes of gene products across all species of organisms in terms of molecular function, cellular component, and biological process. We analyzed gene ontological pathways in a similar way as functional pathway analysis using shared DEGs of CVD and risk factors. For the selection of gene ontologies, we utilized GO biological process, GO molecular function, and GO cellular component databases of Enrichr and did some statistical analysis as well as literature-driven analysis. We identified vasoconstriction (GO:0042310), vascular smooth muscle contraction (GO:0014829), positive regulation of blood coagulation (GO:0030194), positive regulation of heart contraction (GO:0045823), cardiomyocyte differentiation (GO:0035051), cholesterol import (GO:0070508), inflammatory response (GO:0006954), regulation of angiogenesis (GO:0045765), leukocyte aggregation (GO:0070486), positive regulation of chemokine production (GO:0032722), and positive regulation of MAPK cascade (GO:0043410) as the most significant ontological pathways of CVD as shown in Table 5.

### 3.4. Validation

In order to validate our results, we utilized Enrichr's gold benchmark databases OMIM, dbGAP, and DisGeNET using the shared DEGs of CVD, HTN, T2D, HCL, obesity, and aging. For cross-checking the validity, we collected a list of diseases and associated genes employing the common DEGs of CVD and selected risk factors from the above-mentioned databases. After doing a few steps of statistical analysis on the collected data, we got some maladies and we found that our selected risk factors were present there as shown in Table 6. In this way, we validated the genetic relationships of CVD and its risk factors.

## 4. Discussion

In this study, we developed a bioinformatics and system biological pipeline to investigate the association of CVD with its risk factors at the molecular level. We conducted gene expression analysis, PPI analysis, and functional and ontological pathway analysis and achieved some novel insights and relationships utilizing our pipeline. We identified significant genes, hub proteins, and important pathways that may accelerate CVD research in the future.

From gene expression analysis, we were able to detect a significant number of DEGs where 32 DEGs are shared between CVD and HTN, 17 DEGs between CVD and T2D, 53 DEGs between CVD and HCL, 70 DEGs in CVD and obesity, and 89 DEGs are common between CVD and aging. In addition, we observed that some DEGs are shared among multiple risk factors and CVD. The commonly dysregulated genes (both upregulated and downregulated) in CVD and the risk factors indicate that they are interconnected with each other.

After doing PPI and topological analysis, we got 10 highly connected hub proteins as biomarkers. We searched in the global published research work and found that our identified hub proteins are involved in CVD progression. We got an association between formyl peptide receptors (FPRs) and cardiovascular pathologies [46]. Again, formyl peptide receptor's (FPR) subtype FPR2 is involved in atherosclerosis as well as the stimulation of proinflammatory and proresolution responses, and atherosclerosis is linked with inflammatory responses [47–49]. Tumor necrosis factor (TNF) is known as tumor necrosis factor-alpha (TNF-*α*) which is a small cytokine protein that is involved in the development of coronary heart disease through inflammatory response, plaques, and coronary heart disease acute myocardial infarction [50]. The quantity and duration of TNF-*α* expression are considerable factors for the effect of TNF-*α* on the cardiac system [51]. Short-term TNF-*α* expression may respond to stress in the heart, but long-term expression can cause heart decompensation [52]. Excessive concentrations of TNF-*α* in the bloodstream will lead to left ventricular dysfunction [53], cardiomyopathy, and heart failure [54]. Chemokine ligand 8 (CXCL8) belongs to the CXC chemokine family and is an important mediator of inflammation that is associated with cardiovascular injury [55]. An overexpression of this proinflammatory protein may contribute to coronary artery disease and endothelial dysfunction [55]. Cardiac-associated chemokine C-X-C motif ligand-1 (CXCL1) expressed in neutrophils, macrophages, and epithelial cells is a valid proinflammatory factor that performs a role in mediating the infiltration of neutrophils and monocytes/macrophages. CXCL1 may promote cardiac remodeling and fibrosis, as well as be a therapeutic target for the treatment of cardiac fibrosis in cardiovascular disease [56]. Interleukin-1 (IL-1) is an apical proinflammatory mediator in acute and chronic inflammation and a powerful inducer of the innate immune response that assesses in coronary artery disease [57]. IL-1*β* proteins bind to IL-1 receptors, and polymorphisms in IL-1*β* genes have been linked to atherosclerosis and acute myocardial infarction [58]. Vascular endothelial growth factor-A (VEGF-A) is one of the polypeptide proteins and the most established factor in the VEGF family as well as prognostic biomarker in coronary heart disease patients [59]. VEGF-A influences vascular proliferation and permeability, allowing the body to compensate for hypoxia and speed up inflammatory processes [60]. Cytochrome b-245 beta chain gene is part of cytochrome b-245, which is essential for microbicidal oxidase development in phagocytic cells that plays critical roles in the pathogenesis of coronary artery disease [61]; it is also associated with ventricular hypertrophy and arrhythmia [62]. Prostaglandin-endoperoxide synthase (PTGS) is the key enzyme in prostaglandin biosynthesis, and the adverse effects of PTGS inhibitors on the cardiovascular system have been identified [63]. Integrin subunit alpha X (ITGAX) is a heterodimeric integral membrane protein composed of an alpha chain. It is associated with atherosclerosis and leading to cerebrovascular disease [64, 65]. As a consequence of multiple myocardial stressors, it is likely to cause cardiac myocyte (CM) cell loss [66]. C-C motif chemokine receptor 5 (CCR5) and its ligands CCL3 (MIP-1*α*), CCL4 (MIP-1*β*), and CCL5 (RANTES) also associate and contribute to the initiation and progression of atherosclerosis and related cardiovascular diseases [67].

We identified some statistically significant molecular pathways using gene set enrichment analysis that proves strong associations with the mentioned risk factors and CVD. Among them, the “fluid shear stress and atherosclerosis” pathway is linked to the frictional force that flows blood exerted on the endothelial surface of the vessel wall, which acts a central role in atherosclerosis development [68]. The pathway “cardiac muscle contraction” is a complex process that is connected to cardiac myocytes as well as acts as a key pathway in congenital heart disease [69, 70]. The pathway “rheumatoid arthritis” involves high levels of inflammation, and inflammation accelerates the progression of atherosclerosis and heart disease [71]. According to recent research, the molecular pathway “cytokine-cytokine receptor interaction” directly impacts the myocardium and may serve as a biological mediator of cardiovascular disease [72]. “TNF-*α*-activated signal transduction pathways” in the cardiovascular system may contribute to vascular dysfunction, development, and progression of atherosclerosis and adverse cardiac remodeling following myocardial infarction and heart failure [73]. According to evidence, the “IL-17 signaling pathway” influences the development of atherosclerosis [74]. The “chemokine signaling pathway” performs a critical role in the early stages of cardiovascular disease like atherosclerosis or acute ischemia [55]. Cardiac fibrosis describes the inappropriate proliferation of cardiac fibroblasts (CFs), and clinical and experimental evidence suggested that the transformation of CFs could be regulated by the “transforming growth factor-beta (TGF-*β*) signaling pathways” [75]. Researchers have suggested the intracellular “‘mitogen-activated protein kinase (MAPK) signaling” pathway contributes greatly to the pathogenesis of cardiac vascular disease [76]. The pathway “NF-kappa B signaling” functions as dimers and is involved in the development and progression of both inflammation and cardiac and vascular damage and cell survival [77, 78]. In chronic kidney disease, the “AGE-RAGE signaling pathway” may promote CVD [79], and cellular stimuli activate the “PI3K-Akt signaling pathway” that regulates fundamental cellular functions. Moreover, PI3K/AKT pathway participates in heart failure, cardiomyopathy, cardiac hypertrophy, toxin-induced cardiac injury, myocardial injury, myocardial ischemia, and myocardial infarction [80].

After doing gene ontological enrichment analysis, we identified major gene ontological pathways relevant to CVD and the risk factors utilizing the databases of Enrichr. Identified ontological pathways' associations and influences on the cardiovascular disease are emphasized here. One of our gene ontologies, “vasoconstriction,” can cause high blood pressure, which increases the risk of heart disease and stroke [81]. “Smooth muscle contractions” may regulate blood vessel size and contribute to hypertension [82] as well as plays a major role in vascular diseases [83]. In atherosclerosis, the “positive regulation of blood coagulation” is involved [84], and atherosclerosis is the predominant etiology of CVD. The “positive regulation of heart contraction” influences heart function as well as transcriptional (proliferative) responses and thus associated with cardiovascular disease [85]. At cardiac myocyte level, heart failure is associated with “cardiac myocyte differentiation” [86]. “Cholesterol import” exerts a major impact on the development of atherosclerosis [87]. A central role is assumed by the “inflammatory response” in the pathogenesis of heart failure [88]. According to experimental evidence, the “regulation of angiogenesis” is involved in plaque formation [89] and may cause many diseases such as cardiovascular disease and diabetic microvascular complications [90]. Research consistently showed that regardless of disease severity, “leukocyte aggregation” is an independent indicator of future cardiovascular outcomes, and it has been investigated extensively as a biomarker in cardiovascular diseases [91, 92]. Chemokines are small chemotactic cytokines that trigger integrin activation to induce firm arrest of leukocytes on activated endothelium, thus “positive regulation of chemokine production” contributing to the development of atherosclerosis [93]. Furthermore, “positive regulation of MAPK cascade” is a significant factor in the pathogenesis of cardiac and vascular disease [76].

The above discussion suggests that the identified DEGs, hub proteins, and pathways have a significant association as well as influence on CVD. Therefore, it is clear that the selected risk factors (HTN, T2D, HCL, obesity, and aging) have a meaningful correlation in the progression of CVD. The only limitation of our study is that the whole study has been done using different computational tools, so further wet lab (in vitro and in vivo) experiments are required to verify our findings.

## 5. Conclusions

Using computational and bioinformatics approaches, we examined the GEO dataset for CVD, HTN, T2D, HCL, obesity, and aging to identify the genetic association, clarify the relationships, and illustrate our potential finding of CVD and its risk factors. In this research, we identified DEGs, potential biomarkers from PPI analysis, pathways, and ontology mechanisms that demonstrate the associations between CVD and HTN, T2D, HCL, obesity, and aging, as well as provide insights into the pathogenic characteristics. We also checked the validity of our outcomes by utilizing the three gold benchmark databases (OMIM, dbGAP, and DisGeNET). We suggest that our potential biomarkers and results may assist in treatment strategies, drug targets, and diagnostic activities that could reduce the threats of CVD to human health.

## Figures and Tables

**Table 1:** Descriptions of selected datasets with GEO accession number, platform, tissues, and number of samples.

Disease	GEO number	Platform	Tissues	Control samples	Case samples
Coronary artery disease	GSE98583	Affymetrix	Coronary artery cells	6	12
Myocardial infarction	GSE66360	Affymetrix	Endothelial cells	50	49
HTN	GSE703	Affymetrix	Peripheral blood cells	6	14
T2D	GSE26168	Illumina	Blood cells	23	37
HCL	GSE6054	Affymetrix	Circulating monocytes	13	10
Obesity	GSE60403	Affymetrix	Umbilical cord blood cells	8	8
Aging	GSE13712	Affymetrix	Endothelial cells	6	6

**Table 2:** Number of identified DEGs for CVD and its risk factors.

Disease name	GSE number	Raw genes	Differentially expressed genes	Upregulated genes	Downregulated genes
Coronary artery disease	GSE98583	21154	534	87	447
Myocardial infarction	GSE66360	45118	951	718	233
HTN	GSE703	6434	201	122	79
T2D	GSE26168	10457	572	233	339
HCL	GSE6054	45118	1195	538	657
Obesity	GSE60403	45118	1377	816	561
Aging	GSE13712	45118	2693	1046	1647

**Table 3:** The 10 most significant hub proteins.

Protein	Description	Associated risk factor
FPR2	Formyl peptide receptor 2	Upregulated for HTN
TNF	Tumor necrosis factor	Upregulated for HTN
CXCL8	C-X-C motif chemokine ligand 8	Upregulated for aging
CXCL1	C-X-C motif chemokine ligand 1	Upregulated for HTN
IL1B	Interleukin 1 beta	Upregulated for HTN
VEGFA	Vascular endothelial growth factor A	Upregulated for obesity
CYBB	Cytochrome B-245 beta chain	Upregulated for aging
PTGS2	Prostaglandin-endoperoxide synthase 2	Upregulated for HTN
ITGAX	Integrin subunit alpha X	Upregulated for aging
CCR5	C-C motif chemokine receptor 5	Downregulated for HTN

**Table 4:** Significant functional pathways of CVD are also associated in its risk factors.

ID	Pathway	Genes in pathway	Risk factors	*p* value
hsa05418	Fluid shear stress and atherosclerosis	THBD, FOS, MAP2K5, VEGFA, IL1B, TNF, and ICAM1	Obesity, HTN	1.43E-03
hsa04260	Cardiac muscle contraction	ATP1A2, SLC8A1	HCL	1.95E-02
hsa05323	Rheumatoid arthritis	TGFB2, FOS, VEGFA, TGFB2, CXCL8, CCL20, CXCL1, IL1B, CXCL1, TNF, TLR4, and ICAM1	Obesity, HTN, and aging	4E-03
hsa04060	Cytokine-cytokine receptor interaction	IL1RN, CCL20, TNFRSF21, IL1B, ACKR3, CXCL1, CCR5, IL6ST, TNF, CCL13, TGFB2, CXCL8, CCL20, ACKR3, and CXCL1	T2D, HTN, aging	1.83E-03
hsa04668	TNF signaling pathway	NFKBIA, IL1B, CXCL1, PTGS2, TNF, and ICAM1	HTN	2.86E-08
hsa04657	IL-17 signaling pathway	CXCL8, CCL20, CXCL1, NFKBIA, IL1B, CXCL1, PTGS2, and TNF	Aging, HTN	4.88E-07
hsa04062	Chemokine signaling pathway	CCL13, CXCL8, CCL20, CXCL1, NFKBIA, CXCL1, and CCR5	Aging, HTN	4.06E-03
hsa04350	TGF-beta signaling pathway	TGFB2, THBS1	Obesity	3.96E-02
hsa04010	MAPK signaling pathway	TGFB2, FOS, VEGFA, DUSP1, and AREG	Obesity, aging	3.51E-02
hsa04064	NF-kappa B signaling pathway	CCL13, CXCL8, BCL2A1, PLAU, NFKBIA, BCL2A1, IL1B, PTGS2, TNF, TLR4, and ICAM1	Aging, HTN	1.05E-03
hsa04933	AGE-RAGE signaling pathway	THBD, TGFB2, COL3A1, VEGFA, THBD, IL1B, TNF, and ICAM1	Obesity, HTN	4.19E-04
hsa04151	PI3K-Akt signaling pathway	LAMA4, IL2RA, MDM2, THBS1, and VEGFA	Obesity	7.98E-03

**Table 5:** Significant ontological pathways of CVD are also associated in its risk factors.

GO ID	Pathway	Genes in pathway	Risk factors	*p* value
GO:0042310	Vasoconstriction	ADRA1A, SLC8A1	HCL, HTN	2.02E-02
GO:0014829	Vascular smooth muscle contraction	SLC8A1	HCL	1.9E-02
GO:0030194	Positive regulation of blood coagulation	THBD, TLR4, and THBS1	Obesity, HTN	4.71E-04
GO:0045823	Positive regulation of heart contraction	ADM, ADRA1A, and TGFB2	HTN, aging, and obesity	4.71E-04
GO:0035051	Cardiomyocyte differentiation	TGFB2, NRG1	Aging	1.64E-03
GO:0070508	Cholesterol import	APOA1	Aging	2.78E-02
GO:0006954	Inflammatory response	CCL20, NFAM1, TNFRSF21, MMP25, IL2RA, PTGER3, FOS, LYZ, THBS1, IL1B, CXCL1, FPR2, TNF, and TLR4	T2D, obesity, and HTN	6.30E-05
GO:0045765	Regulation of angiogenesis	TGFB2, PTGIS, THBS1, GTF2I, VEGFA, PTGIS, PRKD1, PML, IL1B, and CYP1B1	Obesity, HCL, HTN, and obesity	3.90E-04
GO:0070486	Leukocyte aggregation	IL1B	HTN	1.18E-02
GO:0032722	Positive regulation of chemokine production	IL1B, TNF, and TLR4	HTN	2.59E-05
GO:0043410	Positive regulation of MAPK cascade	CCL13, TGFB2, CCL20, ACKR3, S100A12, ACKR3, FPR2, TNF, and ADRA1A	HTN, aging	1.22E-03

**Table 6:** Validation of the association of CVD with 5 risk factors employing gold benchmark databases.

Risk factors	Genes	*p* value
HTN	HNF4A, THBD, ACSL1, NFKBIA, FPR2, TNF, ADRA1A, and SCN5A	3.18E-02
T2D	MCTP1, PDE4B, PRKCB, and NLRP1	4.01E-02
HCL	RNF144B, PTGIS, KLK2, ADAMTS6, CFTR, PRKD1, SHB, NOL4, NKAIN2, PSD3, SRGAP3, SLIT2, SAMD12, TRPM3, and TBC1D16	1.44E-02
Obesity	TGFB2, HNF4A, RNF144A, ACSL1, QKI, PADI4, VEGFA, COBLL1, SP4, KCNQ3, BLZF1, and CD44	5.34E-03
Aging	RNF144B, VNN1, TGFB2, ANPEP, KYNU, MED12, TF, CXCL8, PHACTR2, DIP2A, HMCN1, and SAMD12	3.51E-03

## Data Availability

The Gene Expression Omnibus (GEO) datasets of this study were collected from the National Center for Biotechnology Information (NCBI) (https://www.ncbi.nlm.nih.gov/geo/), a publicly available repository.
